# Isolation, Identification and Characteristics of *Aeromonas veronii* From Diseased Crucian Carp (*Carassius auratus gibelio*)

**DOI:** 10.3389/fmicb.2019.02742

**Published:** 2019-11-26

**Authors:** Feng Chen, Jingfeng Sun, Zhuoran Han, Xijun Yang, Jian-an Xian, Aijun Lv, Xiucai Hu, Hongyue Shi

**Affiliations:** ^1^Tianjin Key Laboratory of Aqua-Ecology and Aquaculture, Fisheries College, Tianjin Agricultural University, Tianjin, China; ^2^Institute of Tropical Bioscience and Biotechnology, Chinese Academy of Tropical Agricultural Sciences, Haikou, China

**Keywords:** *Carassius auratus gibelio*, *Aeromonas veronii*, 16S rRNA gene, *gyrB* gene, virulence genes, growing characteristics

## Abstract

*Aeromonas* species often cause disease in farmed fish. In the present study, dominant bacteria were isolated from diseased crucian carp (*Carassius auratus gibelio*). Based on this, a bacterial isolate was tentatively named CFJY-623. This isolate was identified as *Aeromonas veronii* based on analysis of its morphological, physiological, and biochemical features, as well as 16S rRNA and *gyrB* gene sequences. Six virulence genes related to pathogenicity including aerolysin, cytotonic enterotoxins, elastase, glycerophospholipid: cholesterol acyltransferase, lipase, and serine protease were identified in this *A. veronii* isolate. The median lethal dosage (LD50) of the CFJY-623 isolate for crucian carp was determined as 1.31 × 10^7^ CFU/mL. Artificial experimental infection showed that the CFJY-623 isolate caused considerable histological lesions in the fish, including tissue cell degeneration, necrosis, and inflammatory cell infiltrating. Drug sensitivity testing showed that the isolate was susceptible to aminoglycosides, carbapenemes, and nitrofurans. Exploring its growing features showed that this isolate exhibited a high level of environmental adaptability. These results provided a scientific basis for the identification of *A. veronii* and treatment for fish infected by this pathogen.

## Introduction

*Aeromonas* spp. are important conditional pathogens, which often cause infection after host injury or stress response (Jeney and Jeney, 1995). These pathogens cause various infections in human beings, such as endocarditis, gastroenteritis, peritonitis, and septicemia (Janda and Abbott, 2010). They are also primary pathogens in farmed fish (Hossain et al., 2019). In recent years, more and more fish diseases are being caused by *Aeromonas* spp., such as *A. caviae* (Thomas et al., 2013), *A. veronii* (Zhu et al., 2016), *A. salmonicida* (Long et al., 2016), *A. hydrophila* (Chandrarathna et al., 2018), *A. sobria* (Yao et al., 2010) and *A. bestiarum* (Esteve and Alcaide, 2009). Among them, *A. hydrophila* had been considered to be the most harmful for aquatic animals, frequently causing haemorrhagic disease in farmed fish (Wang et al., 2017; Marinho-Neto et al., 2019). However, more recently, *A. veroni* have increasingly been infecting fish, with many similar symptoms and histological lesions, compared to *A. hydrophila.*

*A. veronii*, a Gram-negative, rod-shaped bacterium, was originally described by Hickman-Brenner et al. (1987). It is commonly isolated from environmental, clinical, and food samples (Ghenghesh et al., 2008). *A. veronii* strains have increasing been isolated from diseased fish (Hoai et al., 2019; Raj et al., 2019). The clinical symptoms of infected fish commonly comprised ulcer, fin rot/tail rot, abdominal distention, exophthalmia and hemorrhage. However, symptoms are not presented homogeneously across infected fish; different pathogenicity is observed depending on the particular bacterial isolates or strains. Thus far, there is a dearth of research exploring their characteristics such as virulence, growth features, and histological lesions.

Virulence factors are criteria for judging the virulence and toxicity of bacterial pathogens (Gao et al., 2018). Virulence factors and their synergistic effect are key contributors to the pathogenicity of *A. veronii* (Sun et al., 2016). These virulence factors include cytotonic enterotoxins (*act*, *alt*, *ast*), aerolysin (*aer*), polar flagella (*fla*), serine protease (*ser*), elastase (*ahyB*), lipase (*lip*), DNases (*exu*), glycerophospholipid: cholesterol acyltransferase (*gcaT*), and type III secretion system (*ascV*). Surveying the presence of virulence-related factors in clinical *A. veronii* isolates is essential to understand the pathogenesis and epidemiology (Sen and Rodgers, 2004). However, the data and information available in this respect from diseased fish is sparse.

At the same time, fish diseases caused by *Aeromonas* often occur when water quality changes dramatically, which thus might reflect their growth requirements (Xia et al., 2012). It is important to study the growth characteristics of the bacterial isolate in various condition such as across different pH values and temperatures to better understand what constitutes a suitable growth environment for this pathogen.

The crucian carp *Carassius auratus gibelio*, a subspecies of *C. auratus* (Rylková et al., 2010), is a typical freshwater fish in China with commercial value (Zhou et al., 2001). Recently, diseases with characteristic symptoms of hemorrhaging have occurred in cultured *C. auratus gibelio* in a fish farm in Tianjin, China. After isolation of the dominant bacteria from diseased crucian carp, the typical bacterial isolate was identified as *A. veronii*. In the present study, the results of isolation, identification, pathogenicity, virulent genes, and growing characteristics of the *A. veronii* isolate are described.

## Materials and Methods

### Moribund Fish and Bacterial Isolation

**FIGURE 1:**
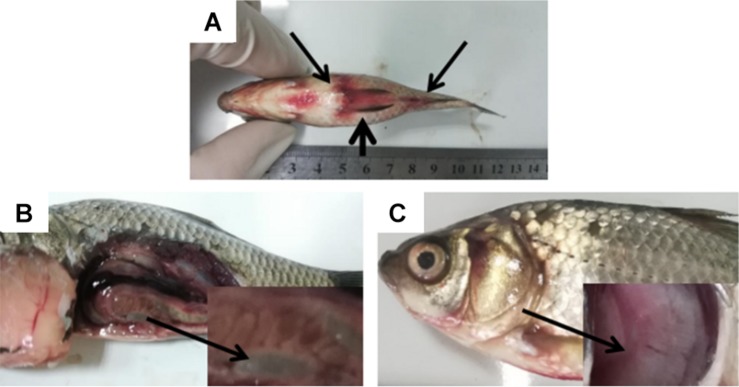
Clinical symptoms in *Carassius auratus gibelio*. **(A)** Abdominal distension (short arrow) and congestion of fin base (long arrow). **(B)** Substantial fluid in the abdominal cavity and intestine (arrow). **(C)** Branchial ischemia (arrow).

Diseased *C. auratus gibelio* were sampled from a freshwater fish farm in Tianjin, China. Moribund fish were taken from the pond to a laboratory at Tianjin Agricultural University. The diseased fish presented obvious clinical signs (Figure 1).

To elaborate, clinical observation on the diseased fish showed abdominal distension and congestion at each fin base (Figure 1A). The abdominal cavities and intestines of the morbid fish were filled with a substantial amount of ascitic fluid (Figure 1B), and the fish gills were pink or gray due to ischemia (Figure 1C). No parasites were found in the diseased fish after examination using inverted microscope. Next, the fish liver, spleen, and kidney were used as samples for virus examination. The liver cell line of the fish was used for virus isolation. The cells were cultured at 20°C in DMEM (Beijing Solarbio Science & Technology Co., Ltd., China) containing 10% fetal bovine serum. No cytopathic effect was observed in the cells, thus excluding viral infection.

Further, three typically diseased fish were used for bacterial collection and further isolation. The fish were dissected after their skin was cleaned with 75% ethyl alcohol. Their liver, spleen and kidney were used for bacterial examination. A Leria-Bertani (LB) medium (10 g/L tryptone, 5 g/L yeast extract, 10 g/L NaCl, 15 g/L agar; pH 7) was employed for bacteria isolation at 30°C for 24 h and the dominant uniform bacterial colonies developed. Some isolates were purified by streaking onto the LB plates three times. A single bacterial colony was selected and inoculated in LB broth at 30°C for 18 h, then preserved at −80°C in the LB medium containing 20% (v/v) sterile glycerol. A dominant strain was tentatively named CFJY-623.

### Physiological and Biochemical Characterization

Morphological observation of the CFJY-623 isolate was carried out by Gram staining. A commercial microtest systems (Hangzhou Binhe Microorganism Reagent Co., Ltd., China) was used for measurement of physiological and biochemical characteristics, including maltose, glucose, sucrose, lactose, fructose, raffinose, xylose, melezitose, sorbose, L-rhamnose, sorbitolum, inositol, dulcitol, adonitol, urea, esculin, Voges-Proskauer test, tartrate, malonate, citrate, starch, salicin, H_2_S, indole, lysine decarboxylase, ornithine decarboxylase, arginine dihydrolase, and motility. Results were observed after incubation according to the manufacturer’s instructions.

### 16S rRNA and *gyrB* Gene Sequences Analysis

The genomic DNA of the CFJY-623 isolate extracted using an UNIQ-10 column DNA extraction kit (Sangon, China) was used as templates for PCR amplification. A pair of universal primer 27F: 5′-AGAGTTTGATCCTGGC TCAG-3′ and 1492R: 5′-GGCTACCTTGTTACGACTT-3′ (Jensen et al., 2002) was used for amplification of the 16S rRNA gene. A pair of primer *gyrB*-F: 5′-GAAGTCATCATGACCGTTCTGCA(TC)GC(TCAG)GG(TCA G)GG(TCAG)AA(AG)TT(TC)GA-3′ and *gyrB*-R: 5′-AGCAGGGTACGGATGTGCGAGCC(AG)TC(TCAG)AC(AG) TC(TCAG)GC(AG)TC(TCAG)GTCAT-3′ (Yamamoto and Harayama, 1995) was used for amplification of the *gyrB* gene. PCR amplification was performed using a Biometra TProfessional Thermocycler (TaKaRa, Japan) according to a previous report (Albarral et al., 2016). The amplified products were observed and then sequenced at Sangon (Shanghai, China). A BLAST search for sequences was carried out via the NCBI website. Phylogenetic trees were established using the neighbor-joining method in the MEGA 5.1 software package following Han et al. (2017).

### Experimental Infections

Sixty healthy *C. auratus gibelio*, with average length 10 ± 1 cm, were kept at 25 ± 1°C with an acclimation period of 14 days. Then, these fish were randomly divided into six groups with each containing ten fish. Following Han et al. (2017), five bacterial concentrations of the CFJY-623 isolate were prepared: 1 × 10^5^, 1 × 10^6^, 1 × 10^7^, 1 × 10^8^ and 1 × 10^9^ CFU/mL. Fish in five groups were subjected to intraperitoneal injection with the five bacterial concentrations at a dose of 200 μL/fish, respectively. The remaining control group was injected with 0.9% physiological saline. All fish were raised for 7 days at 25°C for the purpose of observing and recording pathologic symptoms. The occurrence of disease symptoms and morbidity were also recorded. Bacteria from the liver, spleen and ascites of experimentally infected fish were re-isolated. All interventions were carried out in strict accordance with the guiding principles of the Animal Experiment Ethics Committee at Tianjin Agricultural University.

### Histopathological Observations

Intestine, spleen, liver, kidney and gill from the moribund fish after infection were fixed in Bouin’s fixative, dehydrated in ethanol, embedded in paraffin wax blocks, sectioned at 4 μm, and stained with hematoxylin and eosin (H&E) for histopathological observation.

### Drug Sensitivity Tests

Drug sensitivity tests were performed using drug impregnated disks (Hangzhou Binhe Microorganism Reagent Co., Ltd., China), which contained 34 drugs (Table 1). Four to five disks were placed on LB plates and then inoculated with the CFJY-623 isolate at 30°C for 24 h. A reference *A. veronii* strain ATCC 35624 was also included.

**TABLE 1:** Sensitivity of CFJY-623 to different drugs.

	**Chemicals**	**Disk Content (μg)**	**CFJY-623**	***A. veronii* (ATCC 35624T)**
Penicillins	Oxacillin	1	R	R
	Ampicillin	10	R	R
	Penicillin G	10	R	R
	Amoxicillin	10	R	R
	Piperacillin	100	M	S
Quinolones	Enrofloxacin	5	R	S
	Ciprofloxacin	5	R	S
	Norfloxacin	10	R	S
	Enoxacin	10	R	S
	Nalidixic acid	30	R	S
Cephalosporins	Cefixime	5	R	S
	Ceftazidime	30	R	S
	Cephalexin	30	R	S
	Cefazolin	30	R	S
	Cefoperazone	75	S	S
Aminogly- cosides	Gentamycin	10	M	S
	Neomycin	30	S	M
	Kanamycin	30	S	S
	Amikacin	30	S	S
Carbapenemes	Meropenem	10	S	S
	Imipenem	10	S	S
Nitrofurans	Nitrofurantoin	300	S	S
	Furazolidone	300	S	S
Tetracyclines	Doxycycline	20	R	S
	Minocycline	30	M	S
	Tetracycline	30	S	S
Macrolides	Azithromycin	15	S	R
	Erythromycin	15	R	M
Lincomycins	Clindamycin	2	R	R
Rifamycins	Rifarnpin	5	R	S
Coumarins	Novobiocin	30	R	R
Amphenicols	Chloramphenicol	30	R	S
Glycopeptides	Vancomycin	30	R	R
Polymyxins	Polymyxin B	300	S	S

*S, susceptible; M, moderately susceptible; R, resistant.*

### Screening of Virulence Genes

Eleven virulence genes (*act*, *alt*, *ast*, *aer*, *fla*, *ser*, *ahyB*, *lip*, *exu*, *gcaT*, and *ascV*) were screened by conventional PCR. The primers used for amplification of these genes are shown in Table 2. Each PCR reaction contained 10 μL of 2 × PCR Master Mix (Tiangen Biotech, Beijing Co., Ltd., China), 1 μL of each paired primer, 2 μL of template DNA, and 11 μL of H_2_O. Each PCR reaction commenced with denaturation at 94°C for 2 min, then 30 cycles of amplification, and finally extension at 72°C for 10 min. Each cycle included 94°C denaturation for 30 s, annealing for 50 s and 72°C extension for 30 s. The PCR were conducted using a Biometra TProfessional Thermocycler (TaKaRa, Japan) and the results were recorded after electrophoresis on 1% agarose gel stained with ethidium bromide.

**TABLE 2:** PCR primers, targets, and amplicon sizes.

**Target gene**	**Product size (bp)**	**PCR primers sequence (5′-3′)**	**References**
*act*	232	F:AGAAGGTGACCACCACCAAGAACA	Nawaz et al., 2010
		R: AACTGACATCGGCCTTGAACTC	
*alt*	442	F: TGACCCAGTCCTGGCACGGC	Nawaz et al., 2010
		R: GGTGATCGATCACCACCAGC	
*ast*	331	F: TCTCCATGCTTCCCTTCCACT	Nawaz et al., 2010
		R: GTGTAGGGATTGAAGAAGCCG	
*aer*	431	F: CCTATGGCCTGAGCGAGAAG	Nawaz et al., 2010
		R: CCAGTTCCAGTCCCACCACT	
*fla*	608	F: TCCAACCGTYTGACCTC	Nawaz et al., 2010
		R: GMYTGGTTGCGRATGGT	
*ser*	350	F: CACCGAAGTATTGGGTCAGG	Nawaz et al., 2010
		R: GGCTCATGCGTAACTCTGGT	
*ahyB*	513	F: ACACGGTCAAGGAGATCAAC	Nawaz et al., 2010
		R: CGCTGGTGTTGGCCAGCAGG	
*lip*	382	F: ATCTTCTCCGACTGGTTCGG	Li et al., 2017
		R: CCGTGCCAGGACTGGGTCTT	
*exu*	323	F: AGACATG CACAACCTCTTCC	Nawaz et al., 2010
		R: GATTGGTATTGCCTTGCAAG	
*gcaT*	237	F: CTCCTGGAATCCCAAGTATCAG	Nawaz et al., 2010
		R: GGCAGGTTGAACAGCAGTATCT	
*ascV*	807	F: GCCCGTTTTGCCTATCAA	Li et al., 2017
		R: GCGCCGATATCGGTACCC	

### Growing Characteristics

Growing characteristics were explored according to a previous study (Han et al., 2017). The impact of pH on growth was investigated within a 3–11 value range in LB broth at 37°C; the effect of temperature was assessed at 27°C, 32°C, 37°C and 42°C in LB broth at pH 7. The CFJY-623 isolate was cultured in LB broth until the OD_600_ of the bacterial suspension reached the value of 1.2. This suspension (0.1 mL) was used as inoculation for further determination. The isolate was cultured at 150 rpm and growth was observed for 52 h at 600 nm every 4 h by calculating the optical density.

## Results

### Morphological and Biochemical Characteristics

The CFJY-623 isolate was a typically Gram-negative bacterium. It was rod-shaped, with a length of 1.5 ± 0.3 μm, and grew at 4°C and at 0–4% NaCl concentration (w/v), but not at 5% NaCl. The biochemical characteristics are listed in Table 3. The isolate was motile and urea was hydrolyzed. Further, it registered positive for malonate, the Voges-Proskauer test, and acid production from maltose, arabitol, mannose, glucose, sucrose, lactose, and fructose, but negative for raffinose, xylose, melezitose, sorbose, L-rhamnose, sorbitolum, inositol, dulcitol, adonitol, and production from indole, lysine decarboxylase, and ornithine decarboxylase.

**TABLE 3:** Biochemical characteristics of CFJY-623.

**Characteristics**	**Reaction**	**Characteristics**	**Reaction**
Acid formation from		Tartrate	−
Maltose	+	Hydrolysis of	
Arabitol	+	Esculin	−
Mannose	+	Urea	+
Glucose	+	Voges-Proskauer test	+
Sucrose	+	Production of	
Lactose	+	H_2_S	−
Fructose	+	Indole	+
Raffinose	−	Lysine decarboxylase	+
Xylose	−	Ornithine decarboxylase	+
Melezitose	−	Arginine dihydrolase	−
Sorbose	−	Growth on	
L-rhamnose	−	At 0% of NaCl	+
Sorbitolum	−	At 1% of NaCl	+
Inositol	−	At 2% of NaCl	+
Dulcitol	−	At 3% of NaCl	+
Adonitol	−	At 4% of NaCl	+
Utilization of		At 5% of NaCl	−
Citrate	−	4°C	+
Malonate	+	Motility	+
Salicin	−	Hemolytic	+
Starch	−		

*+, positive; −, negative.*

### Sequence Analysis of the 16S rRNA and *gyrB* Genes

**FIGURE 2:**
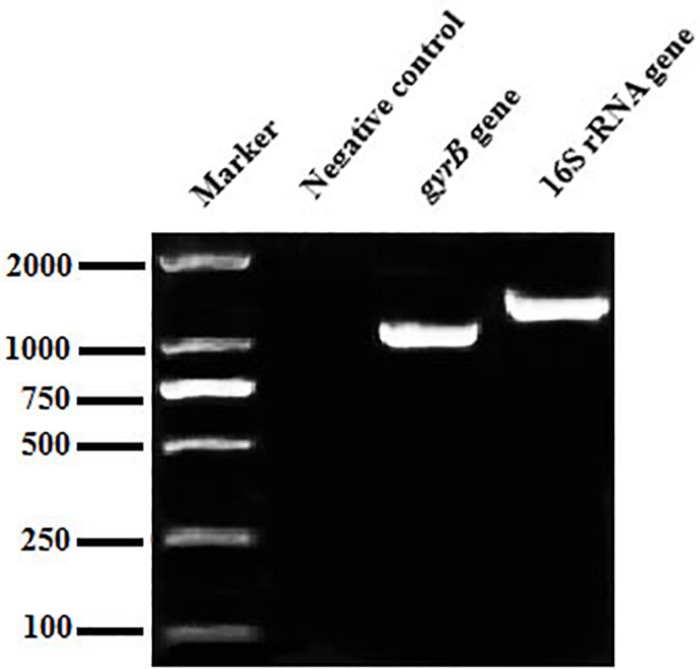
Agarose gel electrophoresis of PCR products of the 16S rRNA and *gyrB* gene of CFJY-623.

**FIGURE 3:**
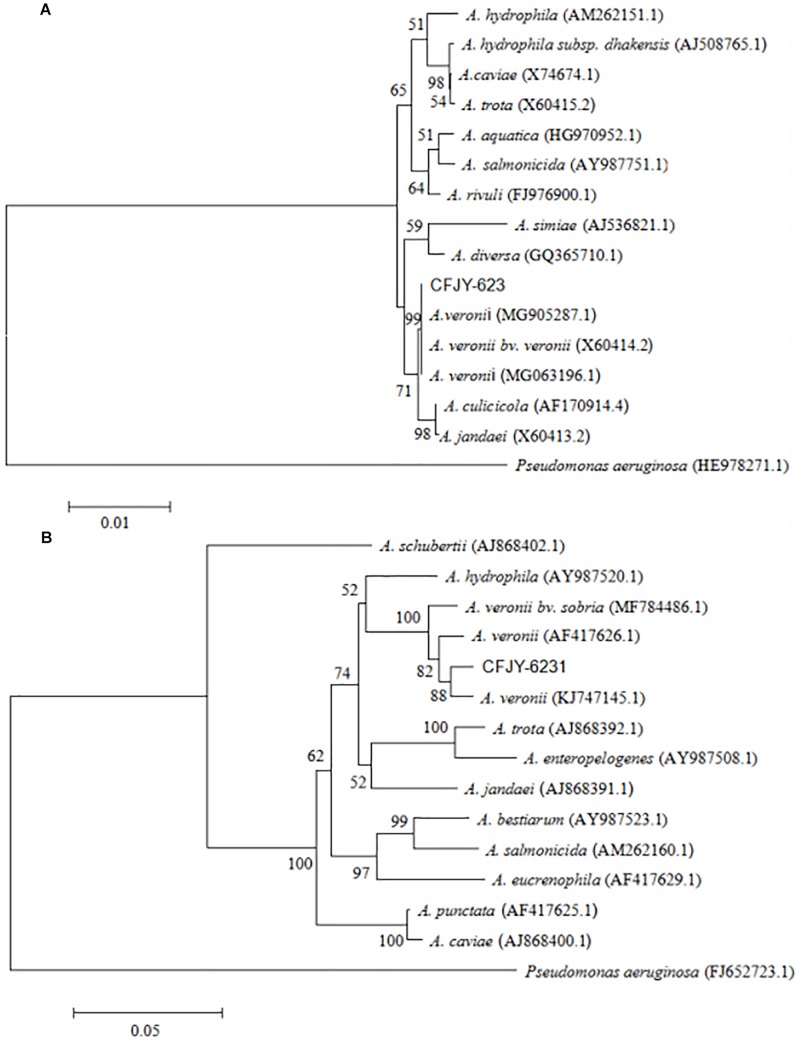
Unrooted neighbor-joining phylogenetic trees based on 16S rRNA gene **(A)** and *gyrB* gene **(B)** sequences of *Aeromonas* strains. Evolutionary history was inferred using the neighbor-joining method. The percentage of replicate trees in which the associated taxa clustered together based on 1,000 bootstrap replicates are shown adjacent to branches. Sequence accession numbers are in parentheses.

The 16S rRNA gene sequence was 1,406 bp in length (Figure 2), with accession number MK392048 in GenBank. It was 100% similar to those of *A. veronii* biovar *veronii* (X60414.2), *A. veronii* (MG063196.1) and *A. veronii* (MG905287.1). The *gyrB* gene sequence was 1,149 bp in length (Figure 2), with accession number MK415380. It was 98–99% similar to those of *A. veronii* biovar *sobria* (MF784486.1), *A. veronii* (AF417626.1) and *A. veronii* (KJ747145.1). Phylogenetic trees were used to understand the relationship between the CFJY-623 isolate and representative *Aeromonas* species (Figure 3). According to phylogenetic trees established on the 16S rRNA sequence (Figure 3A) and *gyrB* sequence (Figure 3B), the CFJY-623 isolate is clearly grouped with a cluster of known species of *A. veronii* strains.

### Experimental Infections

The mortality of fish infected with the CFJY-623 isolate are summarized in Table 4. The values in two groups infected with 1 × 10^9^ and 1 × 10^8^ CFU/mL bacterial suspension are both 100% while those in the other groups are all 10%. The LD50 was calculated as 1.31 × 10^7^ CFU/mL using the Bliss method (Finney, 1985). The symptoms of the artificially infected fish were in good agreement with those of natural illness (Figure 1). Three bacterial isolates were re-isolated from the infected fish and they exhibited the same morphological and biochemical features as the CFJY-623 isolate (data not shown).

**TABLE 4:** Determination of median lethal dosage (LD50) of CFJY-623.

**Concentration (CFU/mL)**	**Dose (μL)**	**Number of tested fish**	**No. of death at different time**				**Mortality (%)**
				
			**12 h**	**24 h**	**36 h**	**2–7 days**	
10^9^	200	10	10	10	10	10	100
10^8^	200	10	9	10	10	10	100
10^7^	200	10	1	1	1	1	10
10^6^	200	10	0	1	1	1	10
10^5^	200	10	0	0	1	1	10
PS	200	10	0	0	0	0	0

### Histopathological Observations

**FIGURE 4:**
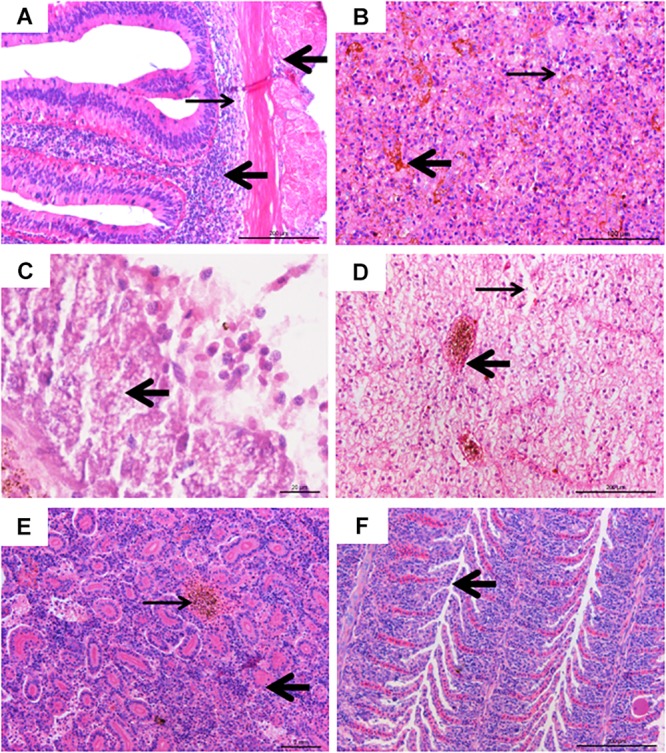
Histological lesions of diseased fish. **(A)** Inflammatory infiltration (thick arrow) and tissue loosening (thin arrow), bar = 200 μm. **(B)** Hemosiderin stratification in spleen tissue (thick arrow), with vacuolar degeneration (thin arrow), bar = 100 μm. **(C)** Islet cell necrosis and vacuolar degeneration (thick arrow), bar = 20 μm. **(D)** Severe intravascular congestion (thick arrow), and severe swelling of liver cells, karyolysis, cell necrosis (thin arrow), bar = 200 μm. **(E)** Renal tubular (thick arrow) necrosis, hematopoietic and inflammatory cell infiltration, interstitial congestion (thin arrow), bar = 200 μm. **(F)** Hyperplasia (arrow) in gill lamellae, with inflammatory cells infiltration, bar = 200 μm.

The experimentally infected fish showed obvious pathological lesions. Multiple organs and tissues displayed extensive hemorrhaging, necrosis, and inflammatory cells infiltrating (Figure 4).

In the intestine, the connective tissue of submucosa was loose and inflammatory cells infiltrated the muscular layer and serosa (Figure 4A). The splenic tissue presented intensive alterations (Figure 4B), with abnormal structure and hemosiderin deposits. Extensive cell necrosis and punctate vacuolation of islet cells were found in the pancreas (Figure 4C). In the liver, severe cell swelling, karyolysis, cell necrosis, and intravascular congestion were observed (Figure 4D). In the kidney, hematopoietic and inflammatory cell infiltration, partial detachment of the tubular epithelium from the basement membrane, and pink chromatin could be observed in the tubular lumen, and there was also noticeable congestion between tubules (Figure 4E). In the gills, mononuclear inflammatory infiltrating, hyperplasia, and necrosis of epithelia, as well as mild bleeding in gill lamellae were observed (Figure 4F).

### Drug Sensitivity

Results of drug sensitivity testing are listed in Table 1. The CFJY-623 isolate was susceptible to cefoperazone, neomycin, kanamycin, amikacin, meropenem, imipenem, nitrofurantoin, furazolidone, tetracycline, azithromycin, polymyxin B. Further, it was moderately susceptible to piperacillin, gentamycin, and minocycline. It was resistant to other tested antibiotics. An *A. veronii* type strain, ATCC 35624, was sensitive to most tested drugs except for oxacillin, ampicillin, penicillin G, amoxicillin, azithromycin, clindamycin, novobiocin, and vancomycin.

### Virulence Genes

**FIGURE 5:**
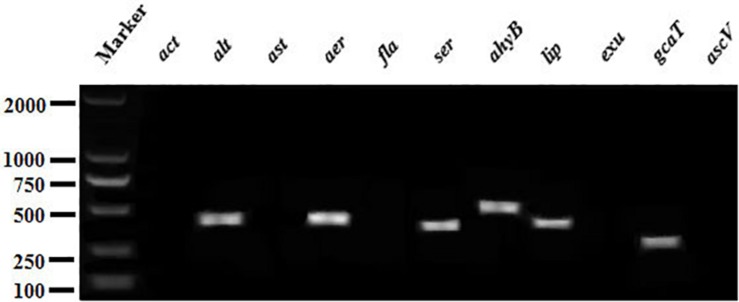
Agarose gel electrophoresis of the amplification products of virulent genes. Six virulent genes (*aer*, *alt*, *ahyB*, *gcaT*, *lip*, and *ser*) were present in CFJY-623.

The PCR profiles of 11 virulence genes screened in this study showed that six genes (*aer*, *alt*, *ahyB*, *gcaT*, *lip*, and *ser*) was present in the CFJY-623 isolate (Figure 5).

### Growing Characteristics

**FIGURE 6:**
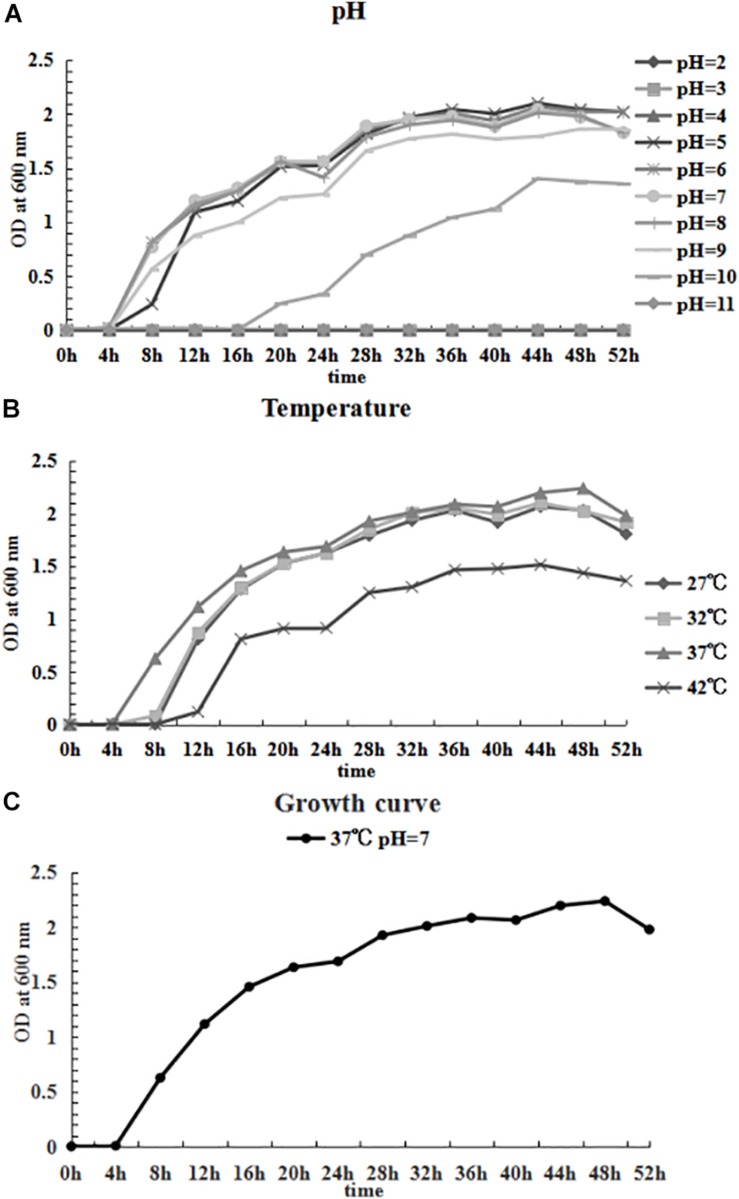
Growing characteristics of CFJY-623. Growth at pH 2-11 **(A)** and temperature 27–42°C **(B)**. The growth curve of CFJY-623 at pH 7.0 and 37°C exhibited four distinct phases of bacterial growth **(C)**.

The growing characteristics of the CFJY-623 isolate were studied (Figure 6). As shown in Figure 6A, the growth and concentration of this isolate were maximized at pH 7. The latent phase of growth was somewhat extended at pH 5, and extended markedly at pH 10. At pH 6, 8 and 9, the growth trends were similar, and considerable final concentrations were obtained. Growth was halted at pH 2, 3, 4, and 11. In terms of temperature, as shown in Figure 6B, growth and concentration were maximized at 37°C. At 42°C growth was clearly inhibited. The growth curve drawn at pH 7 and 37°C (Figure 6C) shows the latent phase occurred between 0–4 h, the logarithmic phase was between 4–20 h, and the stationary phase was between 20–48 h. A secondary growth period was noted during 24–36 h within the stationary phase. After 48 h, the growth curve entered the aging phases.

## Discussion

The genus *Aeromonas* contains a number of opportunistic pathogens causing diseases of aquatic and terrestrial animals, including human beings (Van der Marel et al., 2008). Of which, *A. veronii* has been described as an important pathogen of human beings as well as aquatic animals (Cui et al., 2007). Herein, an *A. veronii* bacterium was isolated from diseased *C. auratus gibelio*.

The biochemical features of the CFJY-623 isolate were basically consistent with those of the *A. veronii* type strain ATCC 25604 (Hickman-Brenner et al., 1987), with positive reactions to lysine and ornithine decarboxylase, negative reactions to arginine dihydrolase, and utilization of sugars including maltose, mannose, glucose, and sucrose. It is worthwhile identifying phenotypic features of bacteria; however, this becomes implausible when only classic phenotypic features are applied (Zhang et al., 2011). Therefore, molecular identification in terms of the 16S rRNA and *gyrB* genes was pursued.

16S rRNA sequencing is one of the most powerful and commonly used methods for identifying bacteria (Busse et al., 1996). However, *Aeromonas* spp. exhibit relatively low discrimination (Zhang et al., 2011) and as such their 16S rRNA gene sequences are not ideal for species identification. The *gyrB* gene is variable and conservative due to its inherent genetic codon usage that allows the DNA sequence to undergo more substitutions without changing the amino acid sequence (Kasai et al., 1998). This gene is thus advantageous for species identification within *Aeromonas* spp. Blast alignments showed that both 16S rRNA and *gyrB* gene sequences of the CFJY-623 isolate shared the highest identities with those of the known *A. veronii* strains. As expected, the phylogenetic trees built based on the sequences of the two genes showed the CFJY-623 isolate clustered with the *A. veronii* strains.

According to the criteria put forward by Mittal et al. (1980), a bacterial strain can be considered as moderately virulent if LD50 values are within the range of 10^6^ to 10^7^ CFU/g fish body weight. In our study, the LD50 of the CFJY-623 isolate was calculated as 1.31 × 10^7^ CFU/mL which thus indicates that it is moderately virulent to *C. auratus gibelio*. Deaths occurring at 12 h after challenge indicated that the infection caused by this pathogen had a short incubation period. Similar phenomena in tongue soles infected with a pathogenic *Shewanella algae* were reported by Han et al. (2017), who noted that the short incubation period resulted in rapid disease progression.

The diseased fish infected with *Aeromonas* species tended to exhibit various clinical symptoms including cutaneous hemorrhage, gill congestion, hyphema, hemorrhages in fins and operculum, abdominal congestion, abdominal swelling, hepatic portal redness, and intestinal swelling. Different *Aeromonas* strains sometimes caused similar clinical symptoms, however sometimes dissimilarities were observed. Even the same *Aeromonas* species could cause divergent clinical symptoms in different fish. In this study, gray gill and intestinal edema occurred in crucian carp infected with the CFJY-623 isolate. However, these symptoms were not manifested following infection with an *A. veronii* isolate in a study reported by Zhang et al. (2017), but were manifested following infection with an *A. militaris* isolate reported by Li et al. (2017). Histopathological observation of the intestine, gill, and viscera of infected fish showed that the *A. veronii* isolate caused obvious histological changes, which were similar to those associated with *A. hydrophila* (Sun et al., 1991; Marinho-Neto et al., 2019) and *A. sobrinus* (Sun et al., 1991). Fish diseases caused by different *Aeromonas* species are difficult to accurately diagnose according to the agent responsible for clinical symptoms and histological lesions, probably because the different disease processes and pathologicities of the pathogens are complex, subsequently causing multifarious levels of infection. The results of the infection experiment also supported the viewpoint that *A. veronii* could probably be considered as an opportunistic pathogen in extensive circumstances (Xia et al., 2012). Further, this pathogen became more pathogenic to fish under stress or in an unfavorable culture condition.

Virulent genes are good indicators for assessing the pathogenicity of a certain microorganism (Senderovich et al., 2012). Of the 11 virulent genes related to *Aeromonas* detected in this study, six genes (*aer*, *alt*, *ahyB*, *gcaT*, *lip* and *ser*) were present in the CFJY-623 isolate. These genes, respectively, code the aerolysin, cytotonic enterotoxin, elastase, acyltransferase, lipase, and serine protease. These six virulent genes have been extensively utilized to investigate the potential pathogenicity of *Aeromonas* species (Aguilera-Arreola et al., 2007). The *aer* gene is found in most *A. veronii* isolates from cultured catfish (Nawaz et al., 2010). The coding product (aerolysin) is found in *A. hydrophila* and plays a key role in the pathogenesis of its infection (Abrami et al., 2003). Aerolysin and cytotoxic enterotoxin are necessary factors for the motile Aeromonads, which are considered potential foodborne pathogens (Chopra and Houston, 1999). These two virulence factors play significant roles in the occurrence of infections (Sha et al., 2002; Soler et al., 2002). It has been reported that the *gcaT*, *lip* and *ser* virulence factors play a coherent, integrated role in affecting the overall pathogenicity of Aeromonad infections (Lee and Ellis, 1990; Pemberton et al., 2006). The *lip* gene, which codes an extracellular lipase, increases the severity of Aeromonad infection by participating in alteration of the host plasma membrane (Pemberton et al., 2006). The enzyme elastase coded by the *ahyB* gene supports infection and colonization by damaging tissue and degrading immune proteins (Song et al., 2018). Several virulent genes present in the CFJY-623 isolate indicate that synergistic effects conferred by combinations of these genes contribute to its pathogenicity. This accords with the observation that combinations of more virulent genes commonly harbored in particularly virulent strains of *Aeromonas* (Li et al., 2011).

In this study, the optima for growth of the CFJY-623 isolate was similar to other strains of *A. veronii* (Hu et al., 2012; Shan et al., 2015). The short latent phase displayed on the growth curve of the CFJY-623 isolate might be related to a short incubation period of fish disease caused by it. Findings with respect to its growth and proliferation occurring under unsuited conditions indicate this *A. veronii* isolate is well adapted to the environment. After its preferred nutrients are exhausted, the bacterium probably use other nutrients to maintain growth (Hu et al., 2012). The secondary growth period during 24–36 h after entering the stationary phase indicated the isolate could use a broad-spectrum of carbon and energy sources as nutrients. The results concerning the optimal growth conditions for the *A. veronii* isolate provide the basis for further studies into this pathogen.

Drug sensitivity testing on the CFJY-623 isolate in this study provide a reference for when antimicrobials may be a potential treatment for *A. veronii* infection. This isolate has developed drug resistance to many tested drugs, especially quinolones and cephalosporins, compared with the type *A. veronii* strain ATCC 35624 and the *A. veronii* strain reported by Vila et al. (2002). In addition, although some drugs such as chloramphenicol, norfloxacin, and ofloxacin are officially banned in aquaculture, the results of the antimicrobial spectrum still contribute to the classification of this microorganism.

In sum, *A. veronii* CFJY-623 was isolated from diseased *C. auratus gibelio*. Its characteristics indicated it had considerable virulence and resulted in acute infection in fish. Since the infection caused by *A. veronii* have commonly occurred in a wide range of hosts in recent years, their pathogenicity and epidemiological characteristics warrant attention by the academic community.

## Data Availability Statement

All data generated for this study are included in the article/supplementary material.

## Ethics Statement

The animal study was reviewed and approved by the Animal Experiment Ethics Committee of Tianjin Agricultural University.

## Author Contributions

FC and ZH designed the laboratory experiments. FC and XY conducted the laboratory experiments, data analyses, and drafted the manuscript. J-AX and AL assisted with the laboratory experiments, data analyses, and contributed to drafting the manuscript. JS, XH, and HS supervised the execution of laboratory experiments and data analyses, and provided a critical review of the final version of the manuscript. All authors read and approved the manuscript prior to submission.

## Conflict of Interest

The authors declare that the research was conducted in the absence of any commercial or financial relationships that could be construed as a potential conflict of interest.
